# One amino acid change of Angiotensin II diminishes its effects on abdominal aortic aneurysm

**DOI:** 10.1042/BSR20182055

**Published:** 2019-05-03

**Authors:** Ya Wang, Yinchuan Xu, Congqing Wu, Hongguang Xia, Yingchao Wang, Jinliang Nan, Jinghai Chen, Hong Yu, Wei Zhu, Peng Shi, Alan Daugherty, Hong S. Lu, Jian’an Wang

**Affiliations:** 1 Department of Cardiology, The Second Affiliated Hospital, Cardiovascular Key Laboratory of Zhejiang Province, College of Medicine, Zhejiang University Hangzhou, Zhejiang, China; 2 Saha Cardiovascular Research Center, University of Kentucky, Lexington, KY, U.S.A.; 3 Department of Biochemistry and Molecular Biology, Zhejiang University College of Medicine, Hangzhou, Zhejiang, China; 4 Department of Physiology, University of Kentucky, Lexington, KY, U.S.A.

**Keywords:** angiotensin II, angiotensin A, aortic aneurysms, hypercholesterolemia, knockout mice

## Abstract

Angiotensin (Ang) A is formed by the decarboxylation of the N terminal residue of AngII. The present study determined whether this one amino acid change impacted effects of AngII on abdominal aortic aneurysm (AAA) formation in mice. Computational analyses implicated that AngA had comparable binding affinity to both AngII type 1 and 2 receptors as AngII. To compare effects of these two octapeptides *in vivo*, male low-density lipoprotein receptor (*Ldlr*) or apolipoprotein E (*Apoe*) deficient mice were infused with either AngII or AngA (1 μg/kg/min) for 4 weeks. While AngII infusion induced AAA consistently in both mouse strains, the equivalent infusion rate of AngA did not lead to AAA formation. We also determined whether co-infusion of AngA would influence AngII-induced aortic aneurysm formation in male *Apoe^−/−^* mice. Co-infusion of the same infusion rate of AngII and AngA did not change AngII-induced AAA formation. Since it was reported that a 10-fold higher concentration of AngA elicited comparable vasoconstrictive responses as AngII, we compared a 10-fold higher rate (10 μg/kg/min) of AngA infusion into male *Apoe^−/−^* mice with AngII (1 μg/kg/min). This rate of AngA led to abdominal aortic dilation in three of ten mice, but no aortic rupture, whereas the 10-fold lower rate of AngII infusion led to abdominal aortic dilation or rupture in eight of ten mice. In conclusion, AngA, despite only being one amino acid different from AngII, has diminished effects on aortic aneurysmal formation, implicating that the first amino acid of AngII has important pathophysiological functions.

## Introduction

The renin-angiotensin system (RAS) plays critical roles in many physiological and pathophysiological functions [1,2]. The major bioactive peptide, angiotensin (Ang)II, exerts its effects through activation of AngII type 1 (AT1) and type 2 (AT2) receptors, with AT1 receptor being the major receptor mediating aortic aneurysmal formation [3–5]. Subcutaneous infusion of AngII at rates of 0.5–2.5 µg/kg/min in mice leads to abdominal aortic aneurysm (AAA) formation, as demonstrated by many investigators [6–10].

We chose to investigate AngA because AngA is an endogenous octopeptide found in human plasma by Jankowski and colleagues, although its concentration is much lower than AngII [11]. However, knowledge about the effect of AngA in physiological and pathological processes is very limited. The sequence of AngII is Asp-Arg-Val-Tyr-Ile-His-Pro-Phe, whereas the aspartate of AngII was replaced by alanine in AngA [11]. Previous studies suggest that higher dose of AngA has a similar vasoconstrictive activity as AngII through AT1 receptor as demonstrated by *in vitro* study or a bolus injection of AngA [11–14]. Since AT1 receptor activation plays a critical role in AngII-mediated aortic aneurysm formation [4,15]. The present study determined whether AngA would have an equivalent effect as AngII on aortic aneurysm formation.

## Materials and methods

### Mice and diets

Twenty male low-density lipoprotein receptor deficient (*Ldlr^−/−^*) mice were purchased from The Jackson Laboratory (Stock #2207; Bar Harbor, ME, U.S.A.) and maintained in individually vented cages (maximally five mice/cage) on a light : dark cycle of 14:10 h. The cage bedding was Teklad Sani-Chip bedding (Cat # 7090A; Harlan Teklad, Madison, WI, U.S.A.). Mice were fed a normal rodent laboratory diet (Diet # 2918, Harlan Teklad; Madison, WI, U.S.A.) and given drinking water from a reverse osmosis system *ad libitum*. During experiments, *Ldlr^−/−^* mice were fed a Western diet (Diet # TD.88137; Harlan Teklad) containing 21% wt/wt (equals 42% calories/calories) saturated fat extracted from milk and 0.2% (wt/wt) cholesterol (0.15% supplemented, and 0.05% from the fat source) 1 week prior to and during 4 weeks of AngII or AngA infusion. Seventy male *Apoe^−/−^* mice were purchased from Beijing Vital River Laboratory Animal Technology Co., Ltd. Mice were housed at a density of fewer than five per cage with a light:dark cycle of 12:12 h at the Department of Cardiology, Second Affiliated Hospital, College of Medicine, Zhejiang University, Hangzhou, P.R. China. These mice were fed a normal rodent laboratory diet and given free access to water during the study. All procedures were performed with the approval of the University of Kentucky (IACUC protocol #2006-0009) or Zhejiang Institutional Animal Care and Use Committee.

### Osmotic mini-pump implantation

AngII (Cat# H-1705) and AngA (Cat# H-6498) were purchased from Bachem. They were infused subcutaneously via Alzet osmotic pumps (Model 2004 or 1004; Durect Corporation, Cupertino, CA, U.S.A.) as described previously [16]. AngII or AngA was dissolved in sterile saline. Mice were sedated with isoflurane and pumps were implanted subcutaneously on the right flank of each mouse. Surgical staples were used to close the incision site immediately.

### Necropsy

Necropsies were performed for mice died prior to the termination of each study. Aortic rupture was defined as observation of blood clots in either the thoracic cavity (thoracic aortic rupture) or retroperitoneal cavity (abdominal aortic rupture).

### Quantification of abdominal aortic aneurysms

At termination, after blood collection, right atrium was cut open, and saline was perfused through the left ventricle to remove blood from the systemic circulation. Subsequently, aortas were dissected and placed in 4% paraformaldehyde (Sinopgarm Chemical Reagent Co., Ltd.) overnight at room temperature. After fixation, periaortic adventitia was carefully removed thoroughly. Maximal outer diameter of the suprarenal aorta was measured *ex vivo* as a parameter for AAA quantification using Image-Pro software (Media Cybernetics Inc.). Definition of an AAA includes (1) aortic rupture of the abdominal aortic region, and (2) maximal outer diameter is at least 1.5 times of the mean maximal outer diameter of mice infused with saline or greater than 1.35 mm if saline group was not available.

### Statistical analyses

Statistical analyses were conducted using SigmaPlot software version 12.5 (Systat Software, Inc.). Comparisons between two groups were performed using unpaired two-tailed Student’s *t*-test for normally distributed variables with equal variance and Mann–Whitney rank sum test for data that did not pass normality test. AAA incidence between groups was analyzed using Fisher Exact test. The *P*<0.05 was considered statistically significant.

## Results

### Equivalent rate of AngA infusion to AngII infusion did not induce aortic aneurysm formation in two hypercholesterolemic mouse models

**Figure 1:**
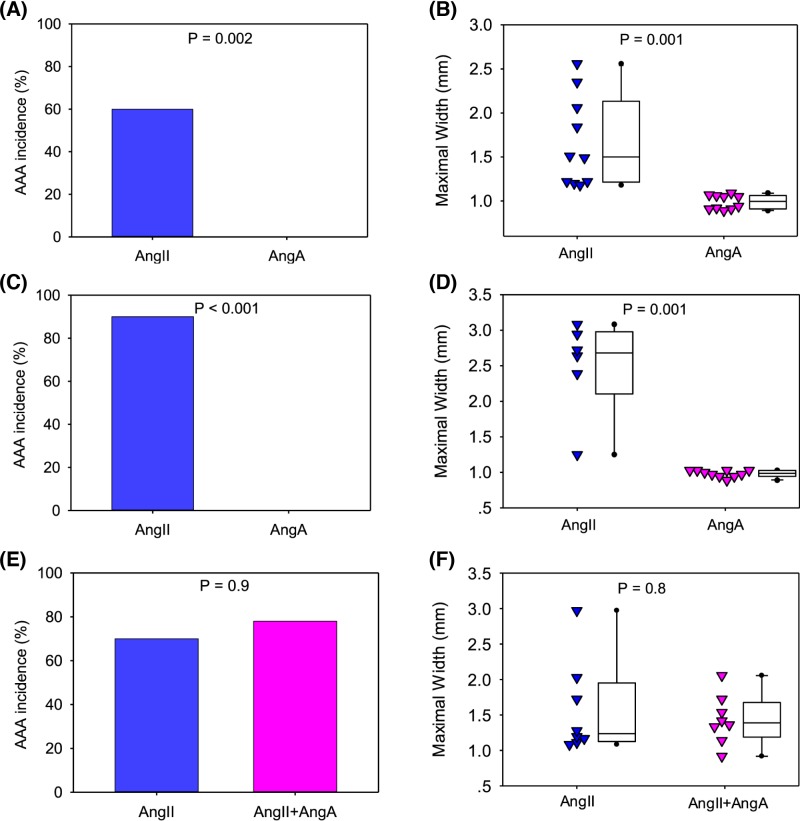
Equivalent rates of AngA infusion to AngII infusion did not influence an AAA in both *Ldlr^−/−^* and *Apoe^−/−^* mouse models Male *Ldlr^−/−^* mice fed a Western diet (**A** and **B**) or *Apoe^−/−^* mice fed a normal diet (**C**–**F**) were infused with AngII (1 μg/kg/min), AngA (1 μg/kg/min), or both AngA and Ang (1 μg/kg/min, respectively) for 4 weeks. Incidence of AAA was defined by calculating ratio (%) of abdominal aortic rupture and AAA formation in each group (A, C, and E) and analyzed by Fisher exact test. Maximum widths of suprarenal aortas (B, D, and F) were measured on *ex vivo* images. Triangles represent values of individual mice (N = 6–10/group). Lines in boxes represent medians, and the boxes span the 25th–75th percentiles, with the bars representing the 5th and 95th percentiles, respectively. Maximal width data were analyzed using Mann–Whitney rank sum test.

There are reports that AngA and AngII had similar affinity to AT1 receptor [11,12]. Our computational analysis also implicated that AngA bound to AT1 and AT2 receptors (Supplementary Figure SI in the online-only Data Supplement). To compare effects of the two octapeptides *in vivo*, we infused AngA versus AngII in either *Ldlr* or *Apoe* deficient mice at a rate of 1 µg/kg/min for 4 weeks. Six of ten mice (60%) had AAA formation in AngII-infused *Ldlr^−/−^* mice. Four mice died of aortic rupture and five of the remaining six mice had AAA formation (AAA incidence: 90%) in AngII-infused *Apoe^−/−^* mice. An equivalent rate of AngA infusion did not lead to aortic rupture or AAA formation in either *Ldlr^−/−^* or *Apoe^−/−^* mice (Figure 1A–D and Supplementary Figures SII and III in the online-only Data Supplement).

There are reports implicating that AngA had AngII-antagonizing effects [11]. We then determined whether AngA would attenuate AngII-induced AAA by infusing AngII alone or co-infusing AngII and AngA at the same rate (1 μg/kg/min) for 4 weeks in male *Apoe^−/−^* mice. Two mice from AngII infusion group died of aortic rupture, one mouse died of aortic rupture in co-infusion group, and the incidence of abdominal aortic aneurysm formation were not significantly different between groups (Figure 1E,F and Supplementary Figure SIV in the online-only Data Supplement).

### A high-rate of AngA infusion had modest effects on aortic aneurysm formation in hypercholesterolemic mouse model

**Figure 2:**
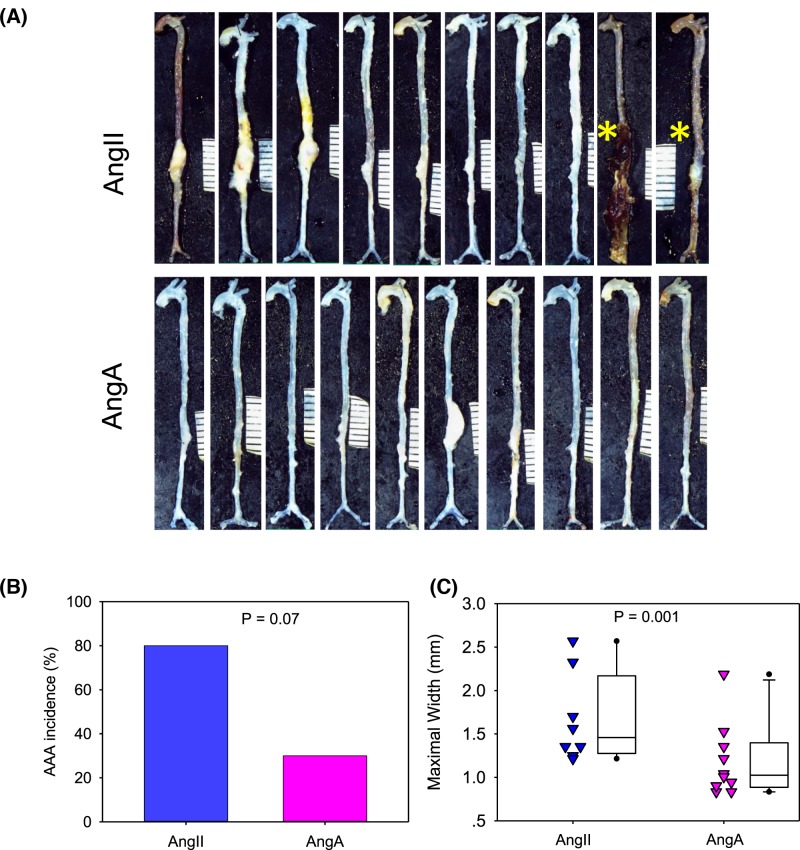
A high rate of AngA infusion had modest effects on aortic aneurysm formation in *Apoe^−/-^* mouse model Male *Apoe^−/−^* mice fed a normal diet were infused with AngII (1 μg/kg/min) or AngA (10 μg/kg/min) for 4 weeks. (**A**) *Ex vivo* aortic images of whole aortas. (**B**) Incidence of AAA was defined by calculating ratio (%) of abdominal aortic rupture and AAA formation and analyzed by Fisher exact test. (**C**) Maximum widths of suprarenal aortas were measured on *ex vivo* images. Triangles represent values of individual mice (N = 8–10/group). Lines in boxes represent medians, and the boxes span the 25th–75th percentiles, with the bars representing the 5th and 95th percentiles, respectively. Data were analyzed by Mann–Whitney rank sum test.

It was reported that a 10-fold higher concentration of AngA had similar vasoconstrictive effects as AngII [11,12]. Therefore, we compared effects of AngA (10 μg/kg/min) infusion with AngII (1 μg/kg/min) infusion on AAA formation in male *Apoe^−/−^* mice. AngII infusion led to aortic rupture in two of ten mice, and six of the remaining eight mice had AAA formation. The higher rate of AngA infusion did not induce aortic rupture, but led to one large AAA and another two mice had minor dilation of the suprarenal region (Figure 2).

## Discussion

In the present study, we found that infusion of AngA with the same rate of AngII did not induce AAA in the two commonly used hypercholesterolemic mouse models. A 10-fold infusion rate of AngA (10 µg/kg/min) led to AAA formation, but with a much lower incidence than AngII at 1 µg/kg/min. On the basis of low plasma ratio of AngA/AngII (∼0.2 in normal subjects and up to approximately 0.8 in patients with end stage of chronic kidney disease), we hypothesize that the pathophysiological processes of AngII decreased after decarboxylation of the Asp [1] to Ala [1] *in vivo*.

Jankowski and colleagues reported that AngA and AngII had similar binding affinity to AT1 receptor in cultured cells [11]. Our computational analysis (Supplementary Figure S1) also predicts that AngA binds to AT1 receptor, which is comparable to AngII binding to AT1 receptor. However, AngA had much less effects on AAA induction, even when its infusion rate was 10-folds of AngII (Figure 2B). This finding implicates that AngA might have much lower affinity to AT1 receptor, although the mechanism is unclear. Indeed, there are also reports that the dose of AngA required to achieve comparable vasoconstrictive effect as AngII was ten times higher than AngII [11,12]. Our future study will aim to define whether the less effect of AngA on AAA is mediated through its lower affinity with AT1 receptor.

Jankowski and colleagues also found that AngA had higher affinity to AT2 receptors than to AngII [11]. Our previous studies have demonstrated that genetic deficiency of AT2 receptor has no effects on AngII-induced AAA development [5]. Hence, the present study does not rule out that AngA may have physiological and pathophysiological functions by binding to AT2 receptor. However, AT2 receptor is abundant during fetal development, but diminishes largely in adults. Therefore, potential physiological or pathophysiological effects of AngA through its binding to AT2 receptor might be modest. AngA is the direct precursor of alamandine, a newly identified member of the RAS, which has several physiological actions that resemble Ang-(1-7), but antagonizes effects of AngII, such as antihypertensive and vasodilation [17]. There is no direct evidence that alamandine or Ang-(1-7) protects against AngII-induced AAA. Taken together, diminished effects of AngII on AAA formation by replacing its first amino acid from aspartate to alanine cannot be explained by its interaction with AT2 receptor or its downstream product alamandine. It remains unclear why a single amino acid change on a not conserved position of AngII would diminish its pathophysiological effects on AAA. It is possible that decarboxylation of the first amino acid aspartate changes the structure of AngII, decreasing the intrinsic activity of binding to AT1 receptor, thereby reducing the harmful effects to cardiovascular system.

The development and progression of AAA involve activation of the RAS [18–20]. One significant impact of our study is the potential to prevent or treat AAA. If AngII can be converted to AngA *in vivo* by enzymatic manipulation, it is possible to prevent or even treat AAA.

## Supporting information

**Supplementary Figure S1:** 

## Abbreviations

AAA: abdominal aortic aneurysm
Ang: Angiotensin
Apoe: apolipoprotein E
AT1: AngII type 1
Ldlr: low-density lipoprotein receptor
RAS: renin-angiotensin system
