# Supplementary material for: One amino acid change of Angiotensin II diminishes its effects on abdominal aortic aneurysm
Source: Biosci Rep. 2019 May 3;39(5):BSR20182055. doi: 10.1042/BSR20182055 (PMC6500891; doi:10.1042/BSR20182055)
Supplement: Supplementary file 1 [file bsr20182055_Supp1.pptx]

## Slide 1
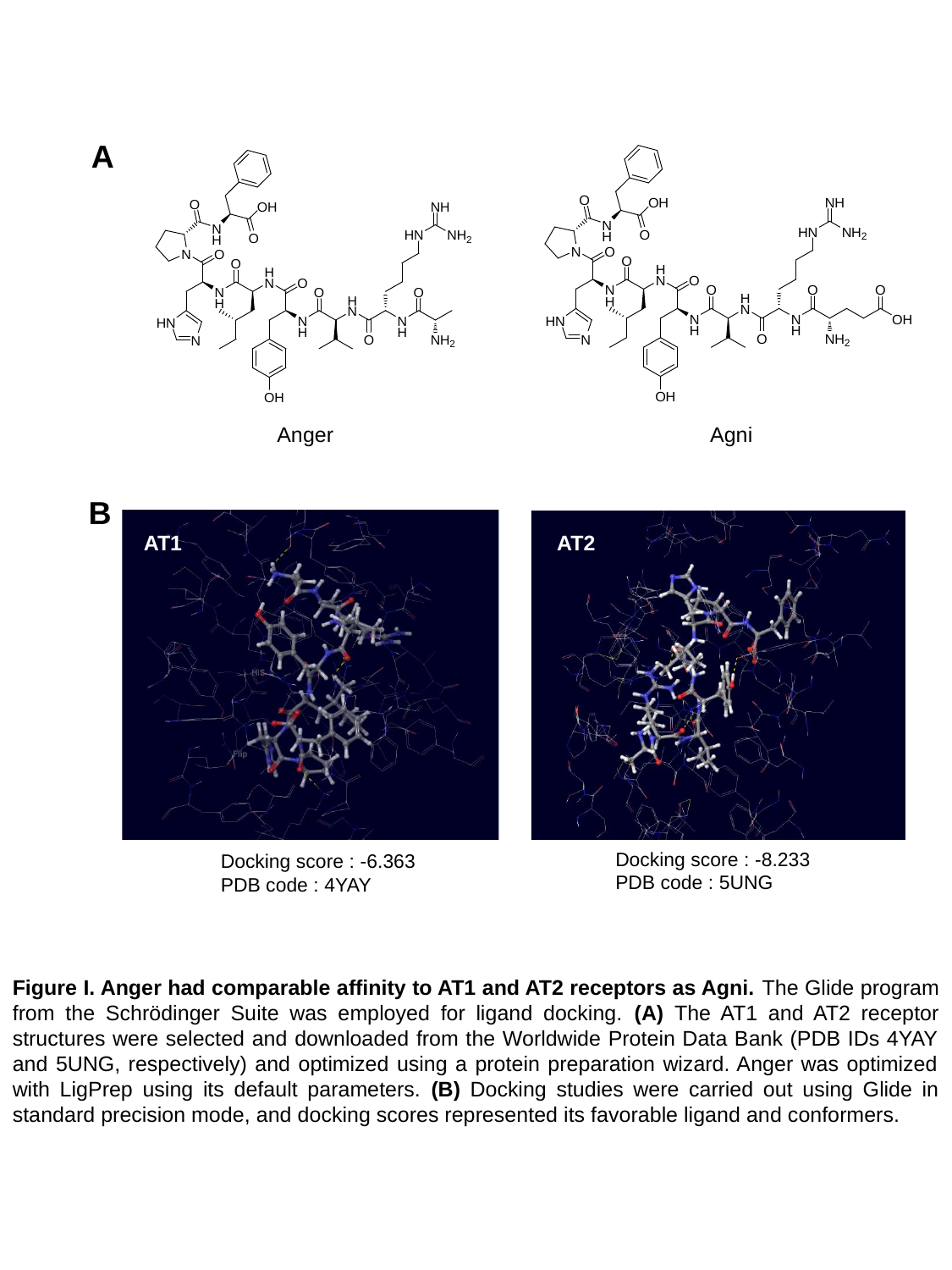

F2-6h
A
Anger
Agni
B
AT2
AT1
Docking score : -8.233
PDB code : 5UNG
Docking score : -6.363
PDB code : 4YAY
Figure I. Anger had comparable affinity to AT1 and AT2 receptors as Agni. The Glide program from the Schrӧdinger Suite was employed for ligand docking. (A) The AT1 and AT2 receptor structures were selected and downloaded from the Worldwide Protein Data Bank (PDB IDs 4YAY and 5UNG, respectively) and optimized using a protein preparation wizard. Anger was optimized with LigPrep using its default parameters. (B) Docking studies were carried out using Glide in standard precision mode, and docking scores represented its favorable ligand and conformers.

## Slide 2
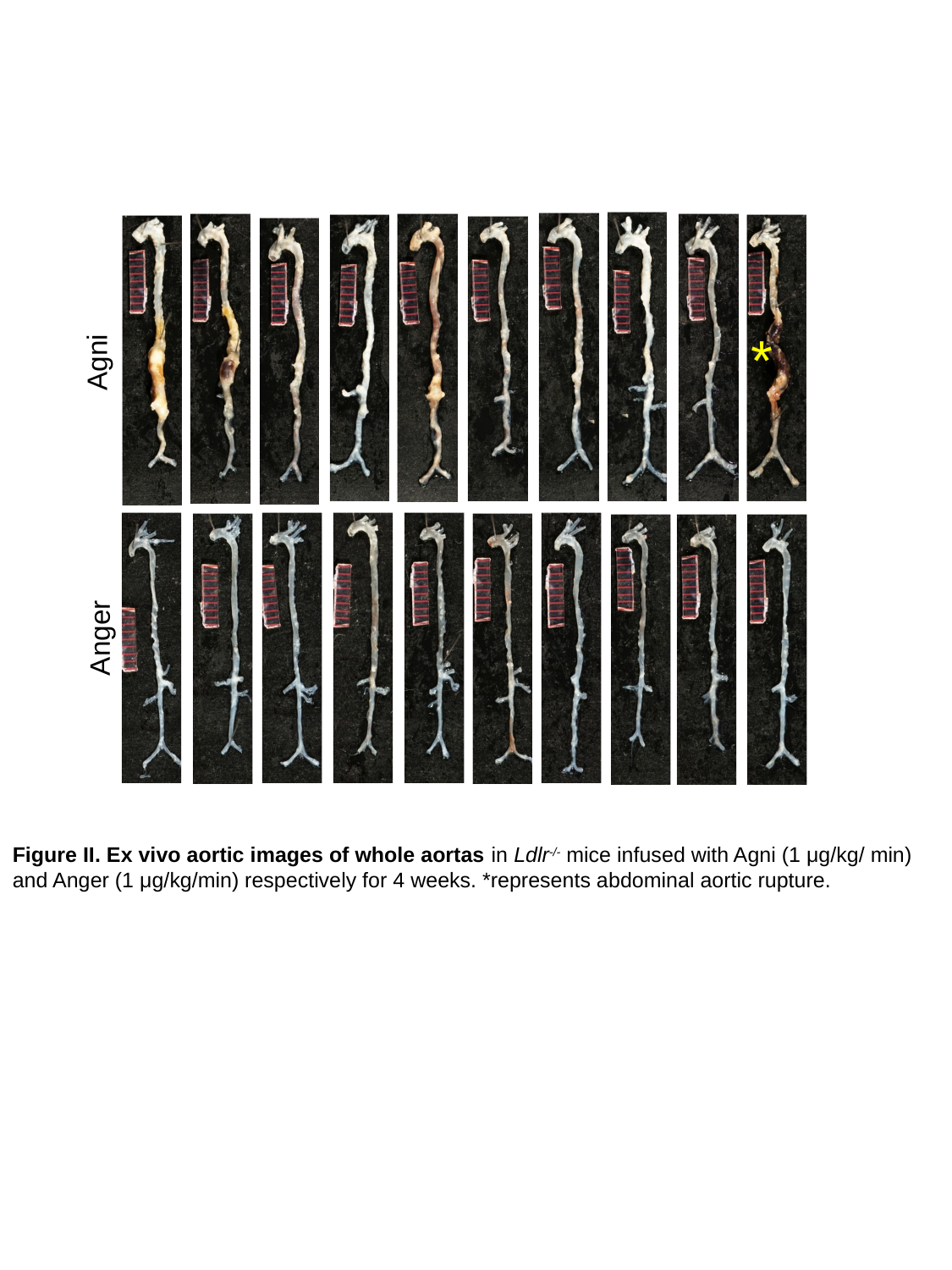

*
Agni
Anger
Figure II. Ex vivo aortic images of whole aortas in Ldlr-/- mice infused with Agni (1 μg/kg/ min) and Anger (1 μg/kg/min) respectively for 4 weeks. *represents abdominal aortic rupture.

## Slide 3
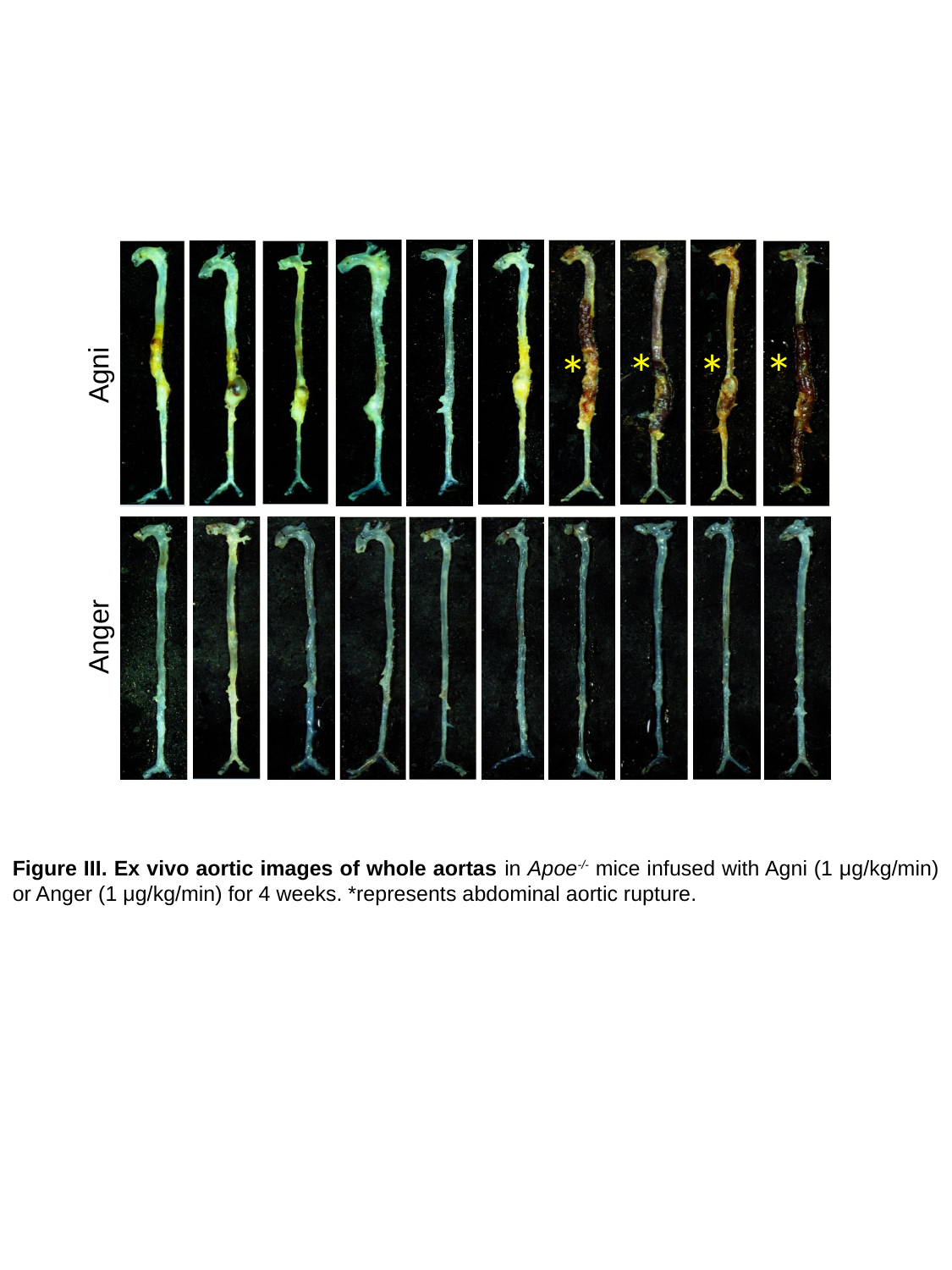

*
*
*
*
Agni
Anger
Figure III. Ex vivo aortic images of whole aortas in Apoe-/- mice infused with Agni (1 μg/kg/min) or Anger (1 μg/kg/min) for 4 weeks. *represents abdominal aortic rupture.

## Slide 4
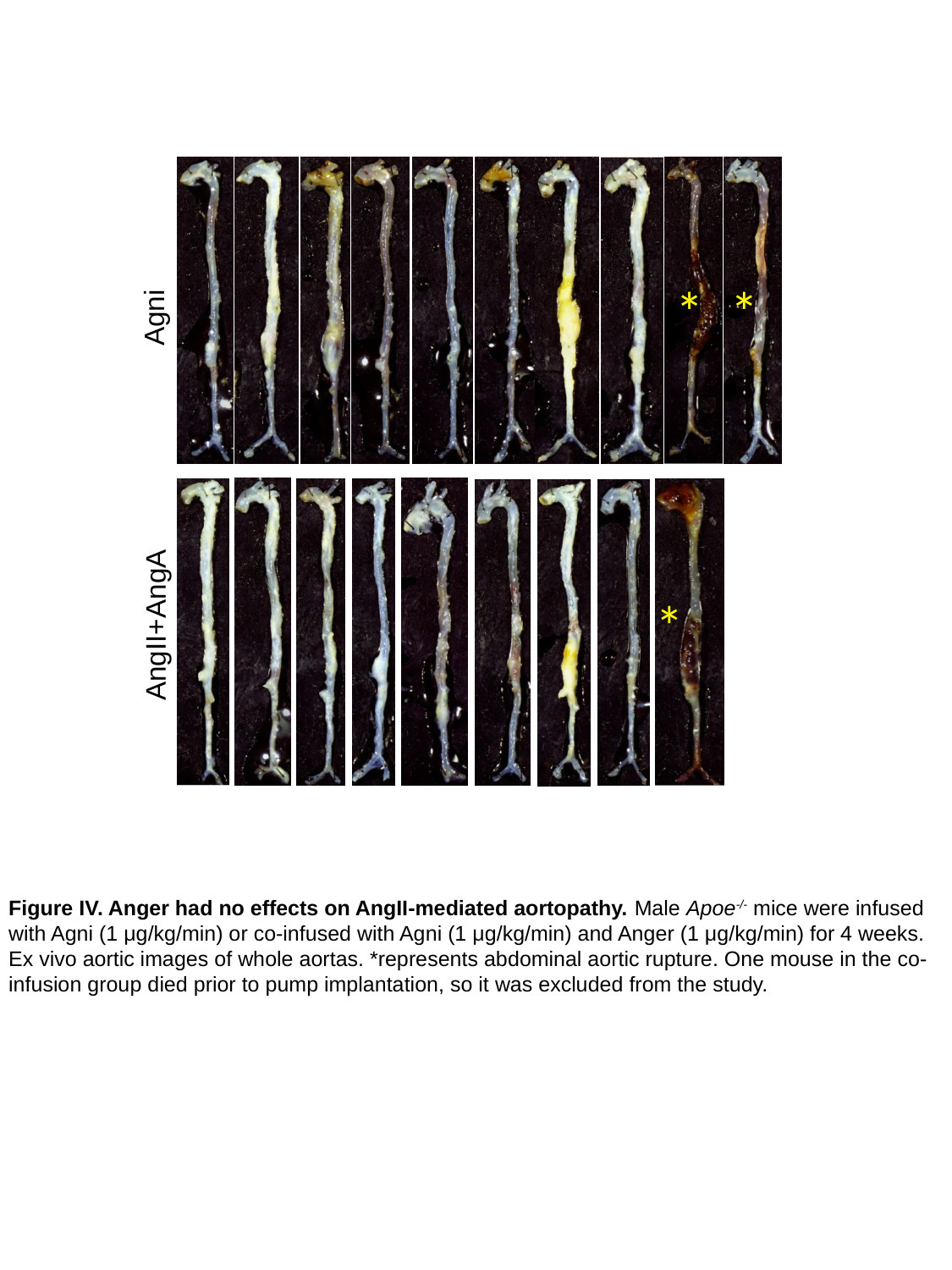

*
*
Agni
*
AngII+AngA
Figure IV. Anger had no effects on AngII-mediated aortopathy. Male Apoe-/- mice were infused with Agni (1 μg/kg/min) or co-infused with Agni (1 μg/kg/min) and Anger (1 μg/kg/min) for 4 weeks. Ex vivo aortic images of whole aortas. *represents abdominal aortic rupture. One mouse in the co-infusion group died prior to pump implantation, so it was excluded from the study.
